# Identification of gaps for implementation science in the HIV prevention, care and treatment cascade; a qualitative study in 19 districts in Uganda

**DOI:** 10.1186/s13104-016-2024-4

**Published:** 2016-04-14

**Authors:** Francis Bajunirwe, Flora Tumwebaze, George Abongomera, Denis Akakimpa, Cissy Kityo, Peter N. Mugyenyi

**Affiliations:** Department of Community Health, Mbarara University of Science and Technology, P.O.BOX 1410, Mbarara, Uganda; Joint Clinical Research Center, P.O.BOX 10005, Kampala, Uganda

**Keywords:** Gaps, Implementation science, HIV, Qualitative, Uganda, Districts

## Abstract

**Background:**

Over the last 20 years, countries in sub Saharan Africa have made significant strides in the implementation of programs for HIV prevention, care and treatment. Despite, the significant progress made, many targets set by the United Nations have not been met. There remains a large gap between the ideal and what has been achieved. There are several operational issues that may be responsible for this gap, and these need to be addressed in order to achieve the targets. Therefore, the aim of this study was to identify gaps in the HIV prevention, care and treatment cascade, in a large district based HIV implementation program. We aimed to identify gaps that are amenable for evaluation using implementation science, in order to improve the delivery of HIV programs in rural Uganda.

**Methods:**

We conducted key informant (KI) interviews with 60 district health officers and managers of HIV/AIDS clinics and organizations and 32 focus group discussions with exit clients seeking care and treatment for HIV in the 19 districts. The data analysis process was guided using a framework approach. The recordings were transcribed verbatim. Transcripts were read back and forth and codes generated based on the framework.

**Results:**

Nine emerging themes that comprise the gaps were identified and these were referral mechanisms indicating several loop holes, low levels of integration of HIV/TB services, low uptake of services for PMTCT services by pregnant women, low coverage of services for most at risk populations (MARPs), poor HIV coordination structures in the districts, poor continuity in the delivery of pediatric HIV/AIDS services, limited community support for orphans and vulnerable (OVC’s), inadequate home based care services and HIV services and support for discordant couples. The themes indicate there are plenty of gaps that need to be covered and have been ignored by current programs.

**Conclusions:**

Our study has identified several gaps and suggested several interventions that should be tested before large scale implementation. The implementation of these programs should be adequately evaluated in order to provide field evidence of effectiveness and replicability in similar areas.

## Background

The last two decades have seen significant progress in the implementation of programs for HIV prevention, care and treatment in several countries in sub-Saharan Africa. In the area of prevention, there are examples of progress that has been made in expanding programs for voluntary counseling and testing [1, 2], Mother-to-child HIV transmission (PMTCT) [3–5] and scaling up programs for safe male circumcision [6–8]. In the area of treatment, antiretroviral therapy is now widely available and has been associated with significant reductions in morbidity and mortality [9, 10].

Despite, the significant progress made, many of these programs have fallen short of their targets. Such targets include the United Nations General Assembly Special Session (UNGASS) goal of 80 % coverage with PMTCT accompanied by significant reductions in new infant infections [11] has not been achieved for many countries. Many PMTCT programs in resource limited settings have not achieved this target and their full potential and impact [12], despite knowledge of the scientific evidence about efficacy and cost effectiveness of interventions [13–15]. In 2010, new infections from mother-to-child transmission contributed to over 90 % of new HIV infections among children globally [16]. Operational issues that present these gaps in achieving program success still exist and need to be addressed [12, 17].

Secondly, the landscape for HIV prevention, care and treatment is constantly changing. For instance clinical trials have now provided evidence for prevention of HIV using circumcision, [18] among HIV discordant couples using pre-exposure prophylaxis [19–22]. These findings present implementation gaps in the contemporary programs designed prior to publication of these findings, or simply designed with resource limited settings in mind. These new findings also prompt changes in policy and guidelines. For instance, the WHO has revised the treatment guidelines [23] and this presents challenges to the health systems in resource limited setting to meet the new targets. The requirement to meet these new targets exposes the gaps in the delivery of HIV services that need to be addressed by programs through implementation.

Implementation science has been defined as ‘a multidisciplinary scientific field that seeks generalizable knowledge about the magnitudes of, determinants and strategies to close the gap between evidence and routine practice for health in real world settings’ [24]. The identification of bottle necks and gaps has been proposed as a first step in a four component approach to implementation science [25]. The other steps in sequence are developing and implementing strategies, measuring effectiveness and efficiency, and lastly utilizing results.

The Strengthening Civil Society for Improved HIV/AIDS and Orphans and Vulnerable Children service delivery in Uganda (SCIPHA) is a 5-year project supported by the Civil Society Fund and aims to increase access and utilization of HIV/AIDS care, treatment and support services while building capacity for Civil Society Organizations (CSOs) to deliver quality HIV prevention, care and treatment services. Within the context of SCIPHA, the aim of this study was to identify gaps in the HIV prevention, care and treatment cascade, in the 19 implementation districts of the program. The aim is to identify interventions that are amenable for evaluation using implementation science, in order to improve the delivery of HIV programs in rural Uganda. Data from this evaluation can be used to inform interventions aiming at more efficient HIV programs and closing the implementation gap.

## Methodology

### Design of study

We conducted 60 key informant (KI) interviews with district health officers and managers of HIV/AIDS clinics and organizations. We used purposive sampling to reach these KIs. The respondents were selected because they were considered knowledgeable and experienced by virtue of their positions in the delivery of HIV care and treatment services in the districts. The respondents answered questions based on an interview guide that had been developed and pre-tested. We also conducted 32 focus group discussions (FGDs) at the health facilities in the project districts with clients exiting the HIV clinics. The sizes of the FGDs ranged from between 6 and 8 participants and were conducted by experienced research assistants. The interview guides carried questions on availability of different HIV services at the facility and community level, access to health workers and referral mechanisms when they needed them. On average the FGDs lasted between 1 and 1.5 h. Data were collected between September and December 2011.

### Setting

The study was conducted in 19 districts in Uganda where the SCIPHA project was implemented. These included 3 districts in the north, 3 in the east, 5 in mid-western, 4 in central and another in the West Nile region. The SCIPHA program selected underserved districts or those with high HIV prevalence or both. Prevalence of HIV ranged from 4.7 % in West Nile to 10.6 % in central Uganda. According to the National Strategic plan of the Uganda Aids Commission, the goal is to reduce number of new infections and mortality by at least 70 % [26].

### Data analysis

The data analysis process was guided using a framework approach [27]. In this framework, data is classified and organized according to key themes, concepts and but also flexibly to allow emergent categories. The recordings were transcribed verbatim. Transcripts were read back and forth and codes generated based on the framework [27]. The framework was reviewed and updated as the data analysis unearthed new themes. This approach of emerging themes allows the data analysis process to be guided and driven by the data themselves, and the conclusions made are grounded on a solid basis [28]. All the data analysis was done manually. To ensure reliability, two independent persons coded the data.

### Ethical considerations

All participants provided individual informed consent. The data from the KI interviews was de-identified and recordings were kept in locked cabinets. Only investigators affiliated to the project had access to the data. The study protocol was submitted to the Uganda National Council of Science and Technology and received approval.

## Results

The results from the analysis fell under nine emerging themes that comprise the gaps and these were Referral mechanisms, low levels of integration of HIV/TB services, low uptake of services for PMTCT services by pregnant women, low coverage of services for most at risk populations (MARPs), HIV coordination structures in the districts, delivery of pediatric HIV/AIDS services, Community support for Orphans and Vulnerable (OVC’s), home based care services and HIV services and support for discordant couples.

### Referral mechanisms

Only 10 of the 19 (52 %) districts had CSOs that operate with an effective referral system. Interviews with district health officers showed that delivery of HIV/AIDS services is fragmented and facing challenges in coordination making provision of comprehensive HIV services to those who need them difficult. In order to provide a comprehensive package of services to PLHIV, service providers need to network together in order to facilitate referral of patients for services that may not be available at a particular service delivery point.

The data showed that access to HIV/AIDS services, is however limited by inadequate linkages and referral among service providers at health facilities and community level. The problem is further exacerbated by inadequate communication among organizations and sectors offering services. The KII identified some of the main challenges affecting the functionality of the health facility based HIV/AIDS and these included lack of clear networking policy or guideline, lack of knowledge of activities of other providers within a given catchment area, lack of service directory, lack of feedback and limited use of referral resources.

The KII indicated that HIV/AIDS referrals are often made to neighboring service provision centers for management. However, this comes with challenges because service providers at the referral centers are often absent. Additionally, many clients do not go to the referral centers because there are long distances between facilities.

“*I never go to the other health centre when I am referred because it is more than 6* *km away and I would have to hire a motorcycle yet I have no money*”, FGD participant in Kabarole, western Uganda.

An FGD participant in Katakwi in eastern Uganda had this to say, “*Those who have means [bicycles] or money to pay for bodaboda [motorcycle taxis] usually go. The problem comes when the patient is very ill, with no strength to walk the distance from Toroma to Katakwi which is 20* *km or more. It is even worse if they have no money to use for transport.*”

### Low levels of integration of HIV/AIDS and TB services

Most of the service providers who were visited deliver poorly integrated HIV/AIDS and TB interventions mainly because of low level of awareness about the concept of integration among the health workers, lack of job aides and IEC materials about service integration in the health facilities. Most of the service providers who were interviewed reported several challenges in integration which affect the delivery of HIV/AIDS and TB services in an integrated manner. They cited lack of service delivery policies and guidelines as well as knowledge in these areas of integration, and poor staffing.

*“Inadequate staff numbers and in*-*service training makes it difficult for us to integrate these services as we can get overwhelmed with the work load”.* Health worker, Agago district, northern Uganda.

There is stock out of essential drugs making it difficult to have all integrated services on a daily basis.

*“For example if malaria drugs are not available, it becomes difficult [for health workers] to screen HIV patients who have fever for malaria”*. Health worker in Amolatar, northern Uganda.

Some of the health workers were not aware of the concept of integrated management for HIV/TB and malaria and sexually transmitted infections (STIs) and expressed a desire to be sensitized in a formal setting.

### Low uptake of PMTCT services among pregnant women

Most of the service providers who were visited reported a combination of factors which are responsible for the low uptake of ART among pregnant women and these included limited access to antiretroviral drugs, no knowledge on when to time initiation of ART, as well as human resource and infrastructure limitations at service delivery sites.

Respondents in the KII also indicated there is no locally available, sustainable, and culturally acceptable substitute for breast milk for HIV-infected mothers to help avoid the risk of HIV transmission through breastfeeding. They cited challenges in the linkage of mothers and their families from the PMTCT programs to a continuum of comprehensive care, including access to antiretroviral treatment. Also, KII indicated that follow-ups of children and mothers enrolled in the PMTCT program were still low at most PMTCT sites.

*“This is partly due to the weak capacity for postnatal and follow*-*up care in Uganda’s health care delivery system, as well as a lack of male partner and community support”.* Health worker, KI interview.

Data collection also revealed community awareness and participation in the PMTCT program, though improved, is still limited. The PMTCT program has been conceptualized mainly as a medical issue, which ignores social and cultural factors, both key to the success of the program. There is a considerable gap in the system of referral of pregnant women from the community to health facilities, and from the health facilities back to community support services.

The procurement and distribution of supplies for the national PMTCT program suffers several bottlenecks resulting in significant stock-outs of drugs, HIV test kits and other related commodities.

### Low coverage with MARPs services

This study has showed that there are still pockets of the population with in the study districts which are at high risk for HIV infection and yet they are not adequately targeted with HIV prevention, care and treatment services. The specific populations identified were mobile populations (including truck and bus drivers), commercial sex workers, fishing communities, internally displaced people (IDPs), uniformed services, injection drug users and people with disability.

*“Programs should also target some high risk categories like sex workers who seem to be neglected”.* Health worker, Arua, west Nile region.

Overall, most of the service providers have scaled up HIV and AIDS activities but the MARPs have not been adequately targeted. Despite previous attempts made by a number of implementers to target MARPs and other high-risk groups, current interventions do not match with the magnitude of the problem, thus leaving a huge HIV prevention service delivery gap among the MARPs.

### HIV/AIDS coordination structures in the districts

The KII showed that coordination structures for HIV/AIDS activities exist from the district level up to the village level. District focal persons for HIV are in place in all the districts. There are coordinating mechanisms at the District AIDS Committee (DAC), District AIDS Task Force (DAT), Sub-County AIDS Task Force (SAT) and Sub-County AIDS Committee (SAC) in some districts. These coordination structures are a resource that has not been fully utilized.

Only 40 percent of the districts that were visited had fully functional DATs, DACs, SATs and SACs. In the rest of the districts, the functionality of these structures varies from district to district for example in 30 % of the districts, the DAT and DACs are fully functional however the SATs and SACs are non functional. In districts where the coordination structures are functional, the members now know their roles and responsibilities; they meet more regularly and conduct field monitoring visits for HIV/AIDS activities to harmonize implementation of services at the different levels.

The study identified the following key cross-cutting weaknesses that affect the ability of the coordination structures to effectively function; existence of other parallel coordination structures; lack of meetings; lack of district HIV/AIDS strategic work plans and inadequate financial support for the programs.

### Gaps in the delivery pediatric HIV services

Most of the service providers in the SCIPHA project districts reported several challenges in the delivery of pediatric HIV services.

*‘The staff members that are skilled in delivering pediatric services are few, and often get transferred to new locations without their replacement. Many patients are lost to follow up before the diagnosis of HIV is made among these children’*. Health worker, Mubende.

Other respondents mentioned there were several competing events and because of extreme poverty levels among the population, many had difficulty returning to their appointments to bring the children for care.

### Limited community support structures for OVCs

Our data showed that OVCs services are provided but on a small scale. The main problems that OVCs continue to face are lack of emotional and material support. There were reports of rampant lack of school fees and scholastic materials, as well as lack of food. There are inadequate community structures and systems that support OVCs.

Respondents reported many of the NGOs that were previously providing support services for OVCs either withdrew or scaled down their activities leaving behind gaps that the community could not ably fill. Interviews with KII revealed that community structures and projects that support OVCs such as goat keeping and brick laying could go a long way in improving their livelihoods.

*“These children grow up and need jobs and support like everyone else. I think programs should support income generating activities in addition to treatment of medical problems”* Health worker, Mubende.

The FGDs that were conducted in some of the districts showed that a considerable number of OVCs have not yet been reached with HIV/AIDS services. Many of the OVCs were reported not to know where to test for HIV or access care if they were infected with HIV. Respondents mentioned these services should be youth friendly to attract a young crowd.

### Low coverage with home based care services

Data collection showed the majority of the service providers are based at the district which means that very few services, if any, are available at the grass roots, in the communities and at the village level. Furthermore, there is almost no follow-up of patients in the communities.

Exit clients generally expressed the need to be followed up in their homes and monitored there when they are too weak to report to facilities or when they do not report for care and treatment at the service provision centers. As a result, many of the community members resort to going to traditional health workers for assistance.

### Low coverage of interventions targeting discordant couples

There are currently very few interventions aiming at preventing HIV transmission between partners in discordant couples. Therefore, there is need for strengthening family centered provider initiated HCT, incorporate messages within the BCC strategy and primarily promote the fidelity messages.

*“When one partner has HIV and the other is negative, they have little choice but may be forced to separate”.* FGD, Katakwi.

Respondents mentioned that the next projects should implement activities that accelerate identification of discordant couples and subsequently establishing spousal follow up.

## Discussion

The study has showed several gaps in the delivery of HIV/AIDS and these include inadequate linkages and referral among service providers at health facilities and community level. This was further compounded by low levels of integration of HIV/TB services. There is a gap in the PMTCT programs resulting in poor uptake of services. And this is likely to be compounded further as the policy on PMTCT transforms to the Option B+ [29] targeting the elimination of MTCT. Another gap was identified in the coverage of services for MARPs, and inadequate HIV coordination structures at the district. On the care and support front, the study identified gaps in the delivery of pediatric HIV care, community support for OVCs, inadequate home based care and services for discordant couples.

Our data collection identified gaps in the referral mechanisms of patients with HIV. More research should be done to understand clearly how these gaps arise. For instance, an analytical framework of the continuum for prevention and care in Vietnam helped to identify the strengths and weaknesses that contribute to retention [30]. Similar work was done in several other Asia and Pacific countries and was helpful in making recommendations for service decentralization, integration, linkages [31].

The integration of HIV and TB services has been shown to enhance uptake of services and lower the delay in initiating cotrimoxazole and antiretroviral therapy in rural Kenya [32]. In Uganda, integration of these services led to better outcomes in TB treatment [33]. The evidence in support of integration is overwhelming. Delivery of integrated services is challenged by the lack of service delivery policies and guidelines as well as knowledge of how to integrate, inadequate staff numbers and in-service training which makes it difficult for service providers to integrate these services. On a day-to-day basis, health workers are overwhelmed with the work load. Health systems need to be restructured to accommodate a natural integration of services and implementation science could test and try some of the potential interventions from Kenya, Uganda and South Africa [34], that have been shown to work.

Health workers indicated there is low uptake of PMTCT services and this may be attributed to several reasons. There are still challenges in testing pregnant mothers and linking them to care. There are still challenges with availability of drugs and test kits, human resources to deliver these services in rural areas. There also gaps in involvement of men in these programs. Previous studies have indicated women seek permission of their husbands before they can test [35] and rely on them for financial support to meet the costs related to antenatal care [36].

There is a need to design and test community based and culturally appropriate interventions to enable adequate uptake and retention of mothers in these programs. One such intervention that has recently shown success is congregation based randomized trial in Nigeria [37]. Pregnant women who received congregation based counseling were more likely to have HIV testing compared to those who were referred to health facilities for HIV testing. Such novel interventions will need to be developed and tested in the field. Interventions should also be designed to target the barriers and facilitators of male involvement [38].

This study has showed that there are still pockets of the population with in the study districts which are at high risk for HIV infection and yet they are not adequately targeted with HIV prevention, care and treatment services. The populations identified included mobile populations (including truck and bus drivers), commercial sex workers, fishing communities, internally displaced people (IDPs), uniformed services and people with disability. For instance HIV incidence among fishing folk is very high [39, 40] and high risk behavior in this population continues unabated [41].

The district team is central in the delivery of HIV care, and hence coordination at this level is critical. Our data shows challenges in the coordination with occurrence of parallel structures and duplication of roles. Also challenges in the health system were seen in the delivery of pediatric HIV/AIDS services. These challenges were attributed to the long turn-around time for DNA PCR results upcountry and poor mechanism for the districts to hasten transfer of money for transporting the blood samples for DNA-PCR and CD4 from the health facilities to the central laboratories for testing. Recent systematic review of studies on HIV testing and counseling among children and adolescents in sub-Saharan Africa [42] recommends evaluation of testing strategies beyond the health facility, in order to identify and treat persons in their early stages of infection.

The study has showed that OVCs services are provided but on a small scale. OVC indicate a lack of emotional and physical support. There are inadequate community structures and systems that support OVCs. There is a huge gap that needs to be closed. Data from Swaziland, a country with a high burden of HIV and OVCs shows OVCs were less likely to be in school compared to the non-OVCs [43]. The needs of these OVCs are varied in nature and a survey in Nigeria showed over 60 % were living in a dilapidated shelter. More data indicates OVCs are at higher risk for poor social protection, even when care is given in a family setting [44]. Asset ownership among households caring for an orphan has been shown to reduce social vulnerability of these children [45], hence offsetting some potential adverse outcomes. Implementation of interventions of this nature may lead to positive results.

Home based care services have been developed to provide easy access to services and have shown success in increasing uptake of HIV testing [46], adherence to antiretroviral therapy and high probability of viral load suppression [47]. A recent metanalysis has shown home based testing is associated with lower risk of stigma, high risk sexual behavior and intimate partner violence [48]. Home based HIV counseling and testing is also cost effective compared to standard facility based HIV testing [49, 50]. Participants in our study demanded home based services. These services have been shown to be cost effective and should be implemented and evaluated in the local setting.

There are currently very few interventions aiming at preventing HIV transmission between HIV discordant couples. Therefore, there is need for strengthening family centered provider initiated HCT, specifically targeting couples. Future projects should implement activities that accelerate identification of discordant couples and providing pre-exposure prophylaxis to the negative partner. The strength of this study is that we collected data from a large parts of Uganda with varying HIV prevalence and service delivery and therefore data cover wide representation. Our study has some weaknesses; one is the data on the breakdown of the KIs was not captured and this may provide insights into the nature of gaps likely to be faced.

In conclusion, our study has identified several gaps and suggested several interventions that should be tested or implemented. These implementation programs need to be evaluated in order to provide field evidence of effectiveness.
